# The Role of Occlusal Appliances in Reducing Masseter Electromyographic Activity in Bruxism

**DOI:** 10.3390/jcm13237218

**Published:** 2024-11-27

**Authors:** Adrian Marcel Popescu, Diana Elena Vlăduțu, Mihaela Ionescu, Daniel Adrian Târtea, Sanda Mihaela Popescu, Veronica Mercuț

**Affiliations:** 1 Department of Fixed Prosthodontics, University of Medicine and Pharmacy of Craiova, 200349 Craiova, Romania; smpopescu@mail.com; 2 Department of Dental Prosthetics, University of Medicine and Pharmacy of Craiova, 200349 Craiova, Romania; veronica.mercut@umfcv.ro; 3 Department of Medical Informatics, University of Medicine and Pharmacy of Craiova, 200349 Craiova, Romania; 4 Department of Dental Technology, University of Medicine and Pharmacy of Craiova, 200349 Craiova, Romania; danielcetate@gmail.com; 5 Department of Oral Rehabilitation, University of Medicine and Pharmacy of Craiova, 200349 Craiova, Romania; sanda.popescu@umfcv.ro

**Keywords:** 3D printed occlusal appliance, sleep bruxism, awake bruxism, surface electromyography, sleep bruxism personal index, number of sleep-bruxism events/h, awake bruxism personal index, masseter activity index

## Abstract

**Background/Objectives**: Bruxism is a masticatory muscle activity, phasic or tonic, with/without teeth contact, that appears in sleep or an awake state. An instrumental technique used to measure the surface electromyographic (sEMG) activity of the masseter muscle is used to diagnose bruxism activity during sleep and while awake. The objective of this study was to compare the variation in bruxism (sleep and awake) indices and masseter activity indices in low sleep bruxism and moderate sleep bruxism before and after wearing an occlusal appliance (OA) for 3 months each night. **Methods**: A clinical interventional study was designed in which subjects diagnosed with sleep bruxism were randomly selected to be included in the study. After the first sEMG recording, two groups were formed: a low sleep-bruxism group (number of sleep-bruxism events/h between 2 and 4) and a moderate sleep-bruxism group (number of sleep-bruxism events/h equal or higher than 4). All subjects received treatment with a 3D-printed occlusal appliance and wore it each night for 3 months, at which point the second sEMG recording was performed. For each participant of this study, a chart was created that included anamnestic data, clinical data, and sEMG data. The data were statistically analyzed with SPSS, using the Mann–Whitney U and Wilcoxon signed-rank tests. **Results**: A total of 21 participants were included in the final analysis, 18 women and 3 men, with a mean age of 24.5 ± 2.7 years. The OA lowered all bruxism indices in the whole group, but clusters analysis showed a significant reduction in sleep-bruxism indices in the moderate sleep-bruxism group, while in the low-bruxism group, the sleep and awake indices varied insignificantly, and the number of sleep-bruxism events/h remained constant. **Conclusions**: The 3D-printed occlusal appliances significantly lowered the sleep-bruxism indices and sleep masseter activity indices recorded with a portable sEMG device in the moderate sleep-bruxism group. The OA lowered the awake-bruxism indices and awake masseter activity indices in the moderate sleep-bruxism group.

## 1. Introduction

Bruxism is a condition characterized by hyperactivity of the masticatory muscles that has received great attention from the dental community [1], as well as from neurologists [2] and specialists in the field of sleep medicine and orofacial pain [3,4]. Bruxism has an increasing prevalence [5], a still unexplained etiopathogenesis [6], and polymorphic manifestations for which dentists are often the first to be consulted [7,8,9]. Bruxism has a prevalence of 39.33% in young people [5] and 22.22% in the general population [10]. Sleep bruxism was observed in 31.01% of students participating in a study compared to awake bruxism, which was observed in 83.72% of the analyzed subjects [5], of which some had both types of bruxism [11], i.e., 14.73% [5].

From an etiopathogenic point of view, occlusal disharmonies [12,13], pathological muscular contraction [8,14], stress [5,6], anxiety [15], sleep structure alterations [16], and genetic factors [17,18] have been considered to affect bruxism.

Occlusal forces in bruxism exceed physiological occlusal forces [19], and both sleep bruxism and awake bruxism have consequences on the teeth, manifesting as tooth wear (attrition, abfraction) [20,21,22] and tooth fractures [23,24,25]. The manifestations of bruxism in the oral cavity are tooth wear [26,27,28,29], dental fractures [30], pulpal complications of tooth wear and fractures [31,32], dental hypersensitivity, occlusal trauma [33], lesions of the lips, cheeks, and tongue [34], exostoses [35], fractures, and destruction of prosthetic restorations [36,37], all this in addition to masseter muscle hypertrophy [27,29] and masticatory muscle pain [1,2,3,38,39].

For a long time, only sleep bruxism had been researched [40,41], although, in 2012, awake bruxism came to the attention of researchers [38,40]. In 2012, an attempt was made to establish an international consensus regarding the definition of bruxism, its diagnostic system, and the consideration of awake bruxism [40]. Regarding the definition of bruxism, the existing definitions from GPT-8 [42], ICSD-2 [43], and OFPG-4 [44] were used. Thus, bruxism was defined as repetitive jaw–muscle activity characterized by clenching or grinding of the teeth and/or by bracing or thrusting of the mandible with two distinct circadian manifestations; it can occur during sleep (indicated as sleep bruxism) or during wakefulness (indicated as awake bruxism). The second consensus regarding the definition and assessment of bruxism was developed during a meeting of members of the Assessment of Bruxism Status group, with bruxism experts from around the globe attending in March 2017 before the 95th General Session and Exhibition of the International Association for Dental Research [45]. According to this consensus on bruxism published in 2018 [46], the new definitions of bruxism are as follows: Sleep bruxism is an activity of the masticatory muscles during sleep that can be characterized as rhythmic (phasic) or non-rhythmic (tonic), and it is not considered a movement disorder or a sleep disorder in individuals considered physically healthy. Awake bruxism is an activity of the masticatory muscles during the awake period that is characterized by repetitive or sustained contact of the teeth and/or by stiffening or pushing the mandible; it can be characterized as rhythmic (phasic) or non-rhythmic (tonic) and is not considered a movement disorder in individuals considered physically healthy [46].

Both definitions of bruxism refer to the motor activity of the masticatory muscles, which may or may not have pathological significance. Svensson [47] continued the reasoning from these definitions and introduced the notions of normo- and patho-bruxism. Therefore, treatment for bruxism should aim at restoring a state of normo-bruxism instead of curing bruxism [47]. Thus, the definition of bruxism evolved from a pathology and sleep disorder to motor behavior with potentially destructive or protective effects [47,48]. Under these conditions, the treatment of bruxism should aim at reducing the activity of the masticatory muscles and repairing the consequences of bruxism in the oral cavity. Several treatment schemes have been proposed over time [49,50,51,52]. Minakuchi, based on a review, suggests that occlusal appliances represent a safe and relatively effective management approach to reducing the frequency and intensity of sleep bruxism based on electromyography [52].

Currently, the preventive approach focused on reducing the destructive effects of bruxism on the stomatognathic system is considered necessary [53,54,55,56,57,58], and occlusal appliances are standard therapy [56]. An occlusal splint or stabilization splint [53,54,55] can be obtained by different methods, from traditional to entirely digital ones [59,60]. As a result of the digitization of the dental laboratory, the occlusal appliances used in bruxism are obtained by digital technologies, among which 3D printing is the most used [59,60]. According to the study by Reymus et al. [61], occlusal appliances obtained by CAD-CAM technology show similar accuracy for milled appliances compared to printed ones.

The International Consensus in 2018 [46] reconsidered awake bruxism, and investigation methods for it were also proposed [9]. In 2024, after a 4-year analysis, a paper written by the most influential researchers in the field of bruxism was published [62,63]. The paper defined the research tools for bruxism and proposed two axes of bruxism evaluation. Axis A is about assessing bruxism status and consequences. First is a subject-based assessment, second is a clinically-based assessment—examiner report that involves a detailed clinical examination to detect intraoral clinical signs of the effects of bruxism, and third is an instrumentally-based assessment—technology report. The technology report consists of the use of devices to establish with certainty the diagnosis of bruxism as the polysomnographic (PSG) analysis for sleep bruxism and the electromyographic (EMG) analysis for awake bruxism [8]. Since polysomnography is a complicated technique that requires a sophisticated laboratory and the observation of the subject to be examined in this laboratory for at least one study night [64,65], in recent years, a series of portable surface electromyography devices have been validated [66,67]. These devices record the action potential of the masticatory muscle above which the device is located (temporal or masseter), store the data, and process them with the help of a software application [66]. Some devices also obtain results such as bruxism personal index (BPI), bruxism time index (BTI), bruxism effort index (BWI), muscle activity personal index (MPI), muscle effort index (MWI), and muscle time index (MTI) [68]. Some of these devices record data for sleep bruxism and awake bruxism for 24 h [68]. Several articles have been published that used such devices for the study of bruxism [56,69,70,71], and a review [66] analyzed these studies and the use of portable sEMG encouraged for the analysis of bruxism with the entire spectrum of masticatory muscle activities (MMA).

It is, therefore, appropriate to ask how the EMG activity of masticatory muscles changes in subjects with sleep bruxism after wearing an occlusal appliance made of photopolymerizable composite material through the 3D printing technique. To our knowledge, there are no studies in the literature to compare the effects of the OA obtained through 3D printing on the electromyographic parameters measured for 24 h with a dia-BRUXO device (dia-BRUXO, Biotechnovation, San Remo, Italy), a one-channel EMGs.

The study aimed to evaluate the effects of wearing a 3D-printed occlusal appliance for three months on the masseter electromyography activity and the sleep and awake-bruxism indices in patients diagnosed with bruxism.

The null hypothesis states no statistically significant change in masseter electromyographic activity and bruxism indices after wearing OA for three months in patients diagnosed with bruxism.

## 2. Materials and Methods

### 2.1. Sample Selection and Ethical Aspects

The recruitment period was October 2022–June 2024. Participants were selected by computer-generated randomization from a larger group of subjects (students and teaching staff at the Faculty of Dentistry diagnosed with probable sleep bruxism after self-reporting and clinical report of sleep bruxism). The sample size was computed using G*Power 3.1.9.7, Heinrich Heine University Düsseldorf, Germany, considering matched pairs comparisons, a significance level α of 0.05, a power 1 − β equal to 0.8, and a medium effect size value (since there are not many data available in the literature, and with an awareness of practical significance), resulting in a study lot of 20 participants. The start period of the study was July 2022, and the follow-up sEMG recordings at 3 months ended in April 2024, while the study ended in October 2024.

The data were collected during the study by downloading from the device dia-BRUXO with the help of specialized software. The study occurred within dental prosthetics, oral rehabilitation, and prosthetics technology disciplines at the Faculty of Dentistry in Craiova. The study was approved by the Ethics Committee of the University of Medicine and Pharmacy of Craiova, no. 156/25 July 2022. The study respected the Declaration of Helsinki. This study was also registered at ISRCTN.com (ISRCTN: ISRCTN80828502). All subjects signed an informed consent form before enrolling in the study. Each subject was informed about the study protocol and the possibility of withdrawing from the study at any time.

### 2.2. Study Design

The study was designed as a clinical interventional study, in which surface electromyographic recording was performed for 24 h in the natural environment before and after wearing a printed maxillary occlusal appliance for 3 months by a group of subjects with sleep bruxism, and later, the collected data were processed and analyzed statistically. The study was conducted according to CONSORT (Consolidated Standards of Reporting Trials) recommendations [72]. The demographic data of the subjects (age, gender), the data related to the sEMG analysis (sleep duration, wakefulness duration, sensor detachment duration), and the number of bruxism episodes reported at the time of sleep were recorded. Sleep bruxism indices (SB-BWI, SB-BTI, SB-BPI), awake-bruxism indices (AB-BWI, AB-BTI, AB-BPI), the total number of clenching episodes, grinding episodes, and other types of movements such as bracing and thrusting, as well as the data related to the activity of the masseter muscle (MMA) during sleep (S-MWI, S-MTI, S-MPI) and during wakefulness (A-MWI, A-MTI, A-MPI), as well as the maximum value of muscle contraction expressed in µVrms were recorded and analyzed.

The study followed a pilot study in which the 3D-printed maxillary splint was tested for 9 weeks on a subject with sleep bruxism [60]. The pilot study describes in detail the protocol used to make the printed occlusal appliance [60].

### 2.3. Variables

The variables analyzed in the study were duration of sleep and wakefulness periods, duration of sensor detachment, number of bruxism events during sleep and awake time, sleep-bruxism indices (SB-BWI, SB-BTI, and SB-BPI), sleep muscle activity indices of the masseter (S-MWI, S-MTI, and S-MPI), awake-bruxism indices (AB-BWI, AB-BTI and AB-BPI), awake muscle activity indices of the masseter (A-MWI, A-MTI and A-MPI) and their variation before and after wearing the OA. The study included a general analysis of the indices but also a detailed analysis of groups, divided according to the number of bruxism events per hour of sleep as follows: a group with low sleep bruxism (min 2–3 events per hour of sleep) and a group with moderate sleep bruxism (4 or more bruxism events per hour of sleep) [73]. An event was defined as an episode of bruxism represented by a clenching lasting over 2 s, three associated grindings, or a mixed episode with clenching and grinding [73].

Considering the more recent acceptance of awake bruxism as an entity [46], there are no studies in the literature regarding the amplitude and duration of muscle contractions specific to awake bruxism. Therefore, the data collected in this study were reported to the sleep-bruxism indices specified in the literature [73].

### 2.4. Occlusal Appliance

The printed OA workflow was presented in an anterior paper [60]. It included classic silicon impressions of both dental arches and occlusion, casting plaster models, mounting the dental casts in semi-adaptable articulators, scanning the casts, OA design in ExoCad, OA printing, and OA processing [60]. OAs were designed as a stability maxillary appliance (Michigan appliance) and 3D printed in a hard ester-based photopolymer resin, NextDent Ortho Rigid Blue (3D Systems, NextDent B.V., Soesterberg, The Netherlands) [60].

### 2.5. sEMG Recordings

The device dia-BRUXO (dia-BRUXO, Biotechnovation, San Remo, Italy) is a one-channel sEMG holter with a highly sensitive amplifier that offers the possibility to record masseter electromyographic activity for 24 h. The device, with small dimensions of 45 mm × 50 mm × 10 mm and a small weight of 16 g, has a particular shape that allows a reproducible positioning of the device on the region of the left masseter, in front of the left ear. Once positioned, the device stays attached to the skin because of a hypoallergic adhesive in the sensor’s solid gel. Signals are detected using the AgAgCl sensors in the bipolar electrodes, positioned with a 22 mm distance between them. The device has a three-stage analog circuit to process the signal, which includes an amplification circuit, an active bandpass filter (between 110 Hz and 550 Hz), and a root-mean-square (RMS) integrator that adapts the information to be digitalized by a 12-bit analog/digital converter (level of discrimination 4096), with an acquisition every 100 mS. The signal is then processed, stored, and interpreted before downloading data into the computer [67].

The device has a lithium battery of 3.7 V and 110 mAh charged with a USB connection. For each use, the device must charge for several hours to function for over 24 h. Before each recording, personal data of the subject are introduced in the software (name, age, gender, address), and also the complaints correlated with bruxism (tooth wear, abfractions, pain of the masticatory muscles, temporomandibular joint disorder or earache, headache, neck muscles pain, neuralgia, tinnitus, vertigo), optional bite usage and other problems like sleep apnea, reflux, and medication. After the recording, the sleep period is introduced in the software, and recording data are downloaded. The software used for dia-BRUXO makes the difference between different kinds of masseter activity during physiologic movements of the jaw in mastication, deglutition, yawning, speaking, and bruxism events, like clenching, grinding, and other activities. Sleep bruxism differentiates from awake bruxism by introducing the exact sleep period in the software. As a result of the software analysis, the report will include % bruxism indices for sleep and awake bruxism (bruxism personal index BPI, bruxism time index BTI, bruxism work index BWI), number of clenching and grinding episodes, and other bruxism activities, as well as masseter muscle activities (MMA) as masseter work index MWI, masseter time index MTI and % masseter personal index MPI. Other data in the report includes muscular power % with a maximum peak expressed as µVrms and the number of swallows [68].

### 2.6. The Study Protocol

The initial step of the study included 38 participants randomly selected from a larger group of students and teaching staff from the Faculty of Dentistry, University of Medicine and Pharmacy of Craiova, who did a positive self-assessment for sleep brux-ism by completing a questionnaire (unpublished results). Participants, 27 women and 11 men, were between 23 and 35 years old, averaging 25 ± 3.2 years old. A dental chart was completed for each subject, and the information collected from the anamnesis and the data obtained after the clinical examination were recorded. Data extracted were related to pain and fatigue of the masseters in the morning, the presence of hypertrophy of the masseters, clinical data about dental fractures, dental wear, the presence of the white jugal line, and other signs related to sleep bruxism (shiny facets, impression in cheek, tongue and lip, Non-Carious Cervical Lesions (NCCL), cracks of the enamel), which helped establish the diagnosis of sleep bruxism.

Afterward, a 24-h sEMG recording was performed for each subject with the dia-BRUXO device. After recording the sEMG, bruxism (sleep or awake) diagnosis was established with certainty, and the severity of sleep bruxism was determined according to the number of bruxism episodes per hour of sleep [73]. Inclusion criteria were age between 18 and 50 years, subjects with certain bruxism, subjects without prosthetic dental restorations, subjects with a maximum of one missing tooth, subjects with changes in the dentition (presence of cracks, dental wear), subjects willing to wear a dental splint, subjects with good oral hygiene. Exclusion criteria were subjects without certain brux-ism, subjects with systemic diseases, subjects wearing an orthodontic appliance, subjects diagnosed with sleep apnea, subjects with sleep bruxism treated with medication, and subjects wearing dental splints already. After this first EMG recording, from 38 subjects identified by self-report and clinical report with sleep bruxism, 13 subjects were excluded for not achieving cut-off criteria for sleep bruxism (number of events/h greater than 2), and only 25 subjects participated in the OA study (20 women and five men).

Of the 25 participants included in the OA study, two groups were defined: a low sleep bruxism group and a moderate sleep bruxism group according to the indices’ values obtained from the first sEMG recording. The cut-off criteria were the number of bruxism episodes (clenching, grinding, other—bracing, thrusting) per hour of sleep. A value of 2–3 episodes of bruxism per hour of sleep represented low sleep bruxism, and a value equal to or higher than four episodes of bruxism per hour of sleep represented moderate sleep bruxism [73]. From these two groups, 4 participants were excluded by not appearing in the last stage of electromyographic recordings. Therefore, the final two groups were the following:

- The group with low sleep bruxism (number of events per hour of sleep between 2 and 4): 10 subjects (9 females and one male);
- The group with moderate sleep bruxism (number of events per hour of sleep equal to or greater than 4): 11 subjects (9 females and 2 males).

**Figure 1:**
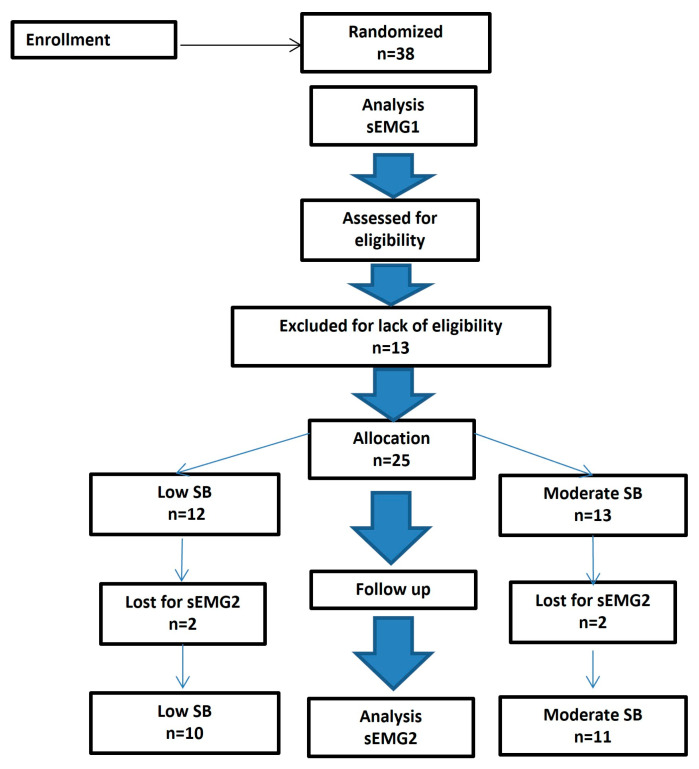
CONSORT diagram (Consolidated Standards of Reporting Trials) with the distribution of subjects within the study.

The detailed steps of the participants’ selection process are described in a dedicated flowchart (Figure 1).

The main inclusion criterion was diagnostic of sleep bruxism, the study aiming to observe the effects of the occlusal appliance on bruxism indices and masticatory muscle activity, the OA being recommended as a treatment for sleep bruxism, but because the portable device permitted registration of masseter electromyographic activity for 24 h, awake-bruxism activity was also monitored since a part of study group subjects had both sleep and awake-bruxism. After the first sEMG recording, the OA was made for each participating subject. Later, OAs were adapted in the oral cavity on the maxillary arch and occlusion. The check-up of the OAs was conducted immediately after application, at 2 weeks and 1 month. Subjects with sleep bruxism received OAs with the recommendation to wear them every night for 3 months. The 24-h sEMG recordings were made initially in the study and 3 months after the application of the occlusal appliance. Since only one sEMG device was used, the study took place for 1 year.

### 2.7. Statistical Analysis

The data collected were initially centralized in Microsoft Excel (Microsoft Corporation, Redmond, WA, USA). Then, data analysis was carried out with SPSS (Statistical Package for Social Sciences) software, version 26 (SPSS Inc., Armonk, NY, USA) between May and July 2024. Continuous variables were described as the pair “mean ± standard deviation” (SD) and median values. Nominal parameters were expressed as frequency distributions and associated percentages. Normality was assessed using the Kolmogorov–Smirnov/Shapiro–Wilk test for all continuous data series. According to the results obtained, comparisons between groups were performed using the Mann–Whitney U test. The Wilcoxon signed-rank test was employed for repeated measurements on the same variables. All *p*-values less than 0.05 represented statistically significant results.

## 3. Results

The sEMG study included 21 participants, 18 women and three men, aged between 23 and 35 years old, with an average age of 24.5 ± 2.7. There are more women than men in the study because women represent most of the dental students in the faculty (over 70%).

**Figure 2:**
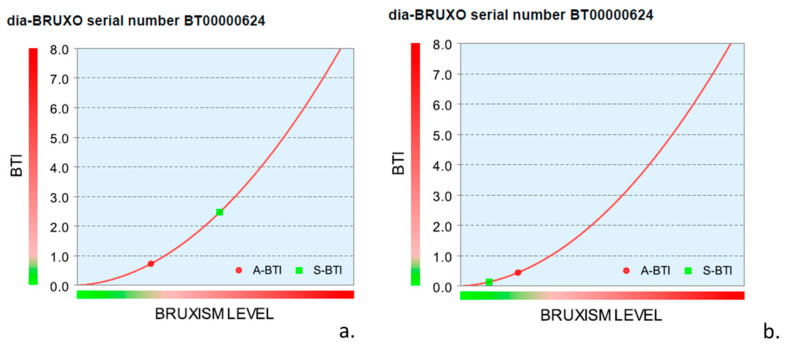
Bruxism level graphic in a subject with sleep bruxism and awake bruxism before (**a**) and after (**b**) wearing an occlusal appliance for 3 months. S-BPI is 2.066% before and 0.133% after OA, and A-BPI is 0.685% before and 0.407% after OA.

Figure 2 shows individual parameters for sleep bruxism and awake bruxism in a graphical image. The evolution of bruxism personal index is evident for this subject with moderate sleep bruxism, who registered a significant drop in bruxism activity after wearing the OA for 3 months (Figure 2).

**Figure 3:**
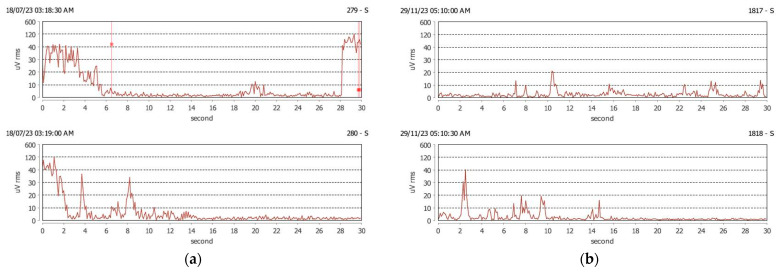
(**a**) Clenching and grinding episodes in a subject with sleep bruxism before wearing OA. The number of sleep-bruxism episodes before OA: 30 clenching episodes and 23 grinding episodes; (**b**) Clenching and grinding episodes in sleep in the same subject after wearing OA. The number of sleep-bruxism episodes after OA: 7 clenching episodes and 0 grinding episodes.

Figure 3 shows the graphical aspect of sleep-bruxism tonic activity (clenching) accompanied by rhythmic (phasic) activity (grinding) before and after wearing OA. For this subject, the number of clenching episodes was lowered, as well as the number of grinding episodes after wearing OA for 3 months (Figure 3).

**Figure 4:**
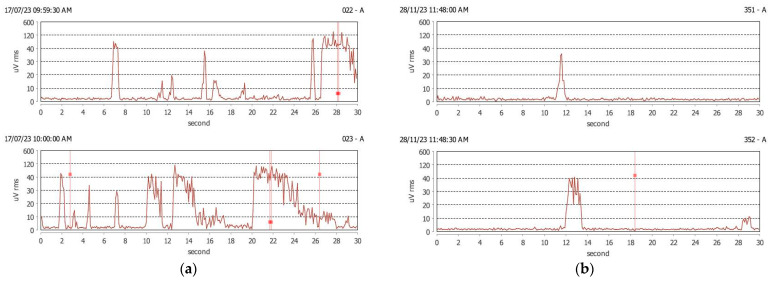
(**a**) Clenching and grinding episodes in a subject with sleep and awake bruxism before wearing OA. Number of awake-bruxism episodes before OA: 106 clenching episodes and 8 grinding episodes; (**b**) Clenching episodes in the same subject after wearing OA. Number of awake-bruxism episodes after OA: 57 clenching episodes and 6 grinding episodes.

Figure 4 shows the graphical aspect of awake-bruxism activity in a subject with awake-bruxism. There are tonic activity (clenching) episodes and rhythmic or phasic activity (grinding) in the awake period, before and after wearing OA. After 3 months of OA, the number of bruxism activity episodes decreased for both types of activity (Figure 4).

Table 1 shows the parameters related to the period of nocturnal sleep and daytime wakefulness, the duration of the sensor detachment, and the number of sleep-bruxism episodes per hour of sleep in the case of the two recordings before and after wearing the OA. For the group with low sleep bruxism, the average hours of sleep were almost seven hours, representing approximately 30% of the recorded period, with more significant variations for the first recording. The duration of sleep was slightly lower in the case of the first recording compared to the second, as was the duration of sensor detachment. Although the number of episodes of sleep bruxism was, on average, the same for the two recordings in the group with low sleep bruxism (2.85), still in the first recording, the variation was smaller than in the case of the second recording, in which the number of episodes of rhythmic sleep bruxism and phase decreased in most participants, but because it increased a lot for one participant, the same average value of the index was reached.

For the moderate sleep bruxism group, the average sleep duration was longer in the first recording compared to the second. The detachment period was also longer in the second recording session than in the first. The number of sleep-bruxism events per sleep hour was higher before the occlusal splint (6.159 ± 2.271) and was lowered almost to half from the initial value after splint wearing (3.370 ± 3.011) (Table 1).

**Figure 5:**
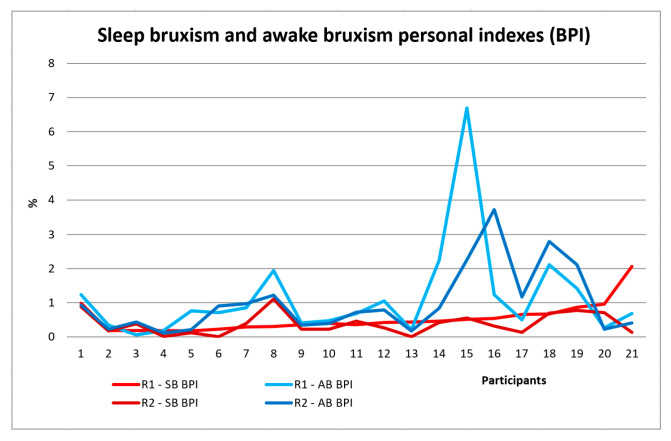
Sleep bruxism (SB) and awake-bruxism (AB) personal indices (BPI) for all study participants before (R1) and after OA (R2).

**Figure 6:**
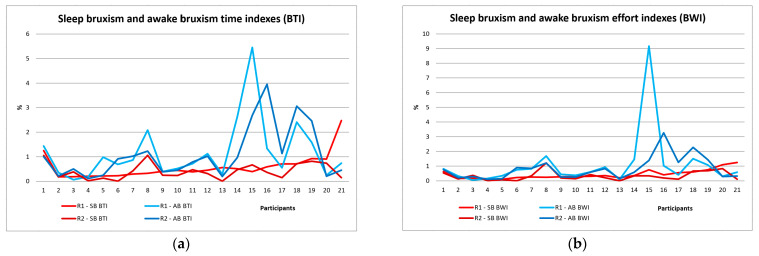
Sleep bruxism (SB) and awake-bruxism (AB) indices for all study participants before (R1) and after OA (R2): (**a**) Bruxism Time indices (BTI); (**b**) Bruxism Work indices (BWI).

Table 2 shows the results of the sleep and awake-bruxism indices, the sleep and awake masseter muscle activity indices, and the number of episodes of rhythmic (phasic) and tonic bruxism for all study participants in the two stages of the study. After the first EMG recording, the sleep bruxism number of events/h in the whole group showed values between 2 and 11, with a mean of 4.583 ± 2.365 number of events/h, more events of clenching (tonic) than events of grinding (phasic) (all participants have sleep bruxism). Sleep-bruxism personal indices recorded SB-BPI varied between 0.178% and 2.066% with a mean of 0.534% ± 0.43%. After OA, these indices decreased, and the mean was 0.38%. Awake-bruxism personal indices AB-BPI varied between 0.063% and 6.687%, with a mean of 1.146% ± 1.418%, and after OA decreased to a mean of 0.998% (Table 2, Figure 5). Sleep-bruxism time indices recorded SB-BTI varied between 0.185% and 2.474% with a mean of 0.587% and decreased to 0.407%. Awake-bruxism time indices AB-BTI varied between 0.064% and 5.449%, with a mean of 1.172%, and decreased to a mean value of 1.093% (Table 2, Figure 6a). Sleep-bruxism work indices recorded SB-BWI varied between 0.10% and 1.249% with a mean of 0.439% and decreased to 0.333%. Awake-bruxism work indices AB-BWI varied between 0.059% and 9.164%, with a mean of 1.093%, and decreased after OA to a mean value of 0.83% (Table 2, Figure 6b).

The producers provided reference values for A-BPI [74]. According to them, A-BPI should be equal to or higher than 1.205% to establish a diagnosis for sleep bruxism and awake bruxism. In the study group, 33.33% of the sleep-bruxism participants had awake bruxism. Also, reference values for other indices were SB-BTI 0.417%, SB-BWI 0.196%, AB-BTI 1.497%, and AB-BWI 0.624%.

A Wilcoxon signed-rank test was conducted to determine the effect of wearing the OA on the bruxism indices. Data are means unless otherwise stated. The differences for all 20 indices were approximately symmetrically distributed, as assessed by a histogram with a superimposed normal curve. For most participants, the indices decreased during the second recording session, following the usage of the OA, compared to the first recording. There was a statistically significant mean decrease for sleep-bruxism indices SB-BWI (from 0.310 to 0.213, *p* = 0.030), SB-BTI (from 0.421 to 0.309, *p* = 0.037), as well as regarding the number of events per hour (from 4.100 to 1.90, *p* = 0.030).

It is worth noting that, for the entire study group, there is a decrease in all the parameters related to sleep bruxism, the sleep muscle activity of the masseter, as well as a decline of the majority of indices of awake bruxism and awake muscle activity of the masseter, except for A-MWI (index of awake bruxism which represents muscle strength during bruxism episodes) and MP (maximum muscle strength), which increases slightly, from 3.571% to 3.63% for A-MWI, and from 318.524 µVrms to 329.714 µVrms for MP. Table 2 also shows the number of subjects in whom increases or decreases of the parameters were recorded in the case of EMG recordings performed after wearing the occlusal appliance. Thus, among the participants, between 61% and 80% showed decreased sleep and awake-bruxism parameters.

For all participants included in the study group, all parameters for sleep bruxism decreased between recordings. Although the OAs were not worn during the day, most parameters for awake bruxism decreased, except for A-MWI and MP. The awareness of the presence of bruxism by the subjects in the study group could explain these results. The results of bruxism indices and muscle activity indices in stages R1 and R2 for the entire group included in the study with sleep bruxism are presented in Table 2 (means and SD are used for ease of presentation).

In the group of subjects with low sleep bruxism (number of events per hour of sleep between 2 and 4), sleep-bruxism indices increased (SB-BWI, SB-BPI), decreased (SB-BTI) or remained constant (2.85 no events/h). Muscle activity indices (S-MWI, S-MTI, S-MPI) for nighttime activity decreased. The awake bruxism indices (AB-BWI, AB-BTI, AB-BPI) and the number of bruxism events (clenching, grinding, others) decreased. The awake muscle activity of the masseter increased (A-MWI, A-MTI, and A-MPI), and the maximum muscle strength (MP) also increased. No statistically significant changes were recorded for the indices compared before and after wearing the OA in participants with low sleep bruxism, *p* > 0.05 (Table 3).

In the group with low sleep bruxism, the values of all indices of sleep bruxism and awake bruxism registered for the first step of the study were in the low range of values for bruxism. From the low sleep bruxism group, 20% of the sleep-bruxism participants had awake bruxism. In the second step of the study, the values of all indices were also in a low range. As for awake bruxism, the number of participants with awake bruxism was lowered to 10%. Masseter activity in this group decreased for sleep bruxism from 2.12% to 1.832% and increased for awake bruxism from 10.242% to 11.566%.

The results of bruxism indices and muscle activity indices in stages R1 and R2 for the group with low sleep bruxism are presented in Table 3.

Within the group of subjects with moderate sleep bruxism (number of events per hour of sleep greater than or equal to 4), sleep-bruxism indices decreased (SB-BWI, SB-BTI, SB-BPI, no events/h), for most of them significantly (*p* < 0.05), except SB-BTI. Muscle activity indices (S-MWI, S-MTI, S-MPI) for nighttime activity decreased. The awake-bruxism indices on work and time (AB-BWI, AB-BTI) decreased. Only the awake-bruxism personal index (AB-BPI) increased slightly, as did the number of clenching bruxism events. The number of awake-bruxism events, such as grinding and others, decreased. The daytime muscle activity of the masseter decreased (A-MWI, A-MTI, and A-MPI), although the maximum muscle strength (MP) increased slightly. In the group with moderate sleep bruxism, the values of all indices of sleep bruxism and awake bruxism recorded during the first step of the study were in a moderate range of values for bruxism (number of events/h of sleep 6.15, with S-BPI of 0.721% and A-BPI of 1.553%). In the moderate sleep bruxism group, 45.45% of the sleep-bruxism participants had awake bruxism. In the second step of the study, the values of sleep-bruxism indices were in a low range of values for sleep bruxism (number of events/h of sleep 3.37, S-BPI of 0.405%), while the values for awake-bruxism indices maintained a moderate range (A-BPI of 1.583%). Awake-bruxism personal index mean value decreased, and the number of participants with awake bruxism was lowered to 36.36%. Masseter activity in this group decreased for both periods of sleep (S-MPI decreased from 3.884% to 3.248%, and A-MPI decreased from 14.067% to 12.59%).

The results of bruxism indices and muscle activity indices in stages R1 and R2 for the group with moderate sleep bruxism are presented in Table 4.

The results of the sEMG recording in the two groups with sleep bruxism (the group with low bruxism and the group with moderate bruxism) before wearing the occlusal appliance are presented in Table 5. According to this table, the participants with moderate bruxism had higher values for all parameters compared to those with low bruxism. The parameters for sleep bruxism (SB-BWI, SB-BTI, SB-BPI, no. of events/h of sleep, clenching), as well as for the sleep muscle activity of the masseter (S-MWI, S-MTI, and S-MPI), showed significant statistical differences between the two groups with sleep bruxism. Although there were substantial differences between the values of awake-bruxism parameters between the two groups, they were not statistically significant.

A comparison of bruxism indices and muscle activity indices in stage R1 between the group with low sleep bruxism and the group with moderate sleep bruxism is presented in Table 5. Participants were divided into two categories: Low sleep bruxism (10 participants, 47.62%) and Moderate sleep bruxism (11 participants, 52.38%).

According to Table 5, participants with moderate levels of sleep bruxism had higher values for all parameters compared to the other participants. A Mann–Whitney U test was run to determine if there were differences in mean dia-BRUXO indices between participants with low and moderate bruxism. Distributions of the indices for both types of bruxism were similar, as assessed by visual inspection. Almost all mean sleep indices were statistically significantly higher in participants with moderate bruxism compared to the other participants, *p* < 0.05. No awake indices were statistically significantly different between participants with low and moderate bruxism, *p* > 0.05 (Table 5).

A comparison of bruxism indices and muscle activity indices in the R2 stage between the group with low sleep bruxism and the group with moderate sleep bruxism is presented in Table 6.

According to Table 6, the values of the indices after wearing the occlusal appliance decreased for participants from both groups with bruxism, more in the group with moderate bruxism, and there are no statistically significant differences between the groups (*p* > 0.05). According to the results, both groups showed bruxism values in the low range after wearing the occlusal appliance.

The comparison of the differences in bruxism indices and muscle activity indices in stages R1-R2 between the group with low sleep bruxism and the group with moderate sleep bruxism are presented in Table 7.

Table 7 emphasizes that the evolution of dia-BRUXO indices between R1 and R2 presented different sleep and awake-bruxism trends for the study participants. For sleep indices, only the number of grinding events differed less between recordings for moderate bruxism than low bruxism. However, for awake bruxism, several parameters presented a reduced evolution between recordings for moderate bruxism compared to low bruxism: AB-BWI, AB-BTI, and AB-BPI, and the number of clenching and grinding events. Statistically significant differences in the evolution between recordings were identified only for the number of clenching events during sleep, as participants with moderate bruxism presented a higher median reduction for these events (median value 21.00) compared to participants with low bruxism (median value 6.00), *p* = 0.013.

**Figure 7:**
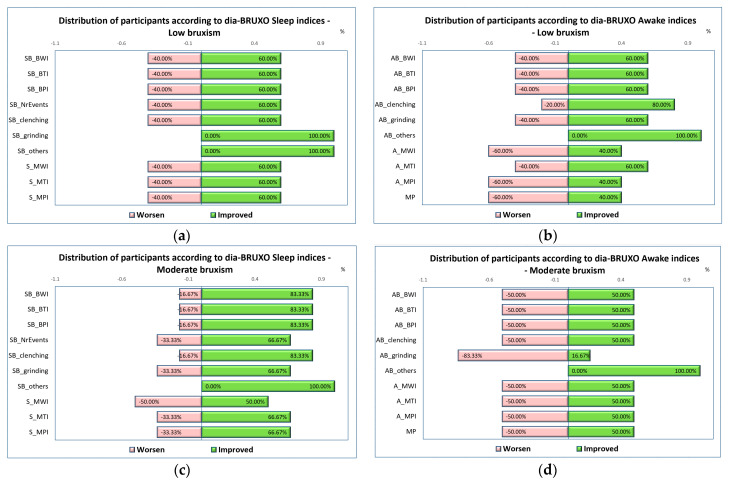
Distribution of participants according to their indices’ evolution and bruxism level: (**a**) Low bruxism, sleep indices; (**b**) Low bruxism, awake indices; (**c**) Moderate bruxism, sleep indices; (**d**) Moderate bruxism, awake indices.

For a better understanding of the distribution of sleep-bruxism indices after wearing the occlusal appliance, a diagram is shown in Figure 7, which presents the number of participants with worsened and improved indices, following the use of the OA, divided by bruxism type and sleep/awake indices.

## 4. Discussion

The present study investigated the effects of 3D-printed occlusal appliances on sleep and awake-bruxism indices recorded with a surface portable electromyograph, comparing subjects with low and moderate sleep bruxism. The OA positively influenced the masseter muscle activity in the group with moderate sleep bruxism, producing a significant reduction in the bruxism indices values for both sleep and awake bruxism. Although some positive effects on the EMG signals were observed in the group with low sleep bruxism, they were not statistically significant. The 3D-printed OAs reduce the masseter muscle activity both at night when they are worn and during the day when they are not worn. The explanation is that the patient probably controls himself better because wearing the splint every evening helps him be aware of bruxism during the daytime.

Although over time, several authors considered that the effects of treatment with OA in bruxism had controversial results [75,76], in a recent systematic review, Ainoosah S. et al. concluded that the therapy with adjustable full-occlusion OA was effective in reducing episodes of sleep bruxism, improving the symptoms reported by the patients and the general state of health [59]. Electromyographic recordings of masticatory muscles can provide valuable data regarding the effectiveness of occlusal splints in bruxism [77]. In a case–control study, the effects of three occlusal stabilization splints (hard, soft, semi-soft) on masseter EMG values and ultrasonographic measured dimensions of masseter muscles were evaluated in 51 individuals with bruxism and 17 controls. The study’s results showed that all three occlusal splints determined a decrease in EMG activity values of both masseter muscles after 3 months of wearing the splint, the change being more significant in the hard occlusal appliance group. Also, mean muscle thickness was greater after wearing the occlusal appliance, with the most significant change in the hard acrylic occlusal appliance group [77]. Their results correlate with the present study results, which showed that OAs lowered most bruxism indices for both types of bruxism (sleep and awake), except for the maximum muscle force, which increased slightly, probably because of the change in muscle thickness. OAs effect was influenced by the severity of bruxism since the changes were statistically significant in the group with moderate sleep bruxism.

Lei et al. [78] investigated the effects of two occlusal appliances with full occlusal coverage and one previously modified OA on individuals diagnosed with bruxism. The previously modified splint demonstrated increased comfort and greater effectiveness in reducing occlusal force and electromyographic activity of the anterior temporalis and masseter muscles. Bergmann et al. [79] showed that full-coverage biofeedback occlusal appliances effectively reduced sleep-bruxism episodes. These results were consistent with previous research and the idea that OA may be an effective therapeutic intervention in reducing sleep bruxism activity [80] and the number of sleep-bruxism episodes [81].

Occlusal appliances can change over time (abrasion) due to contact with the teeth during bruxism’s clenching and grinding movements [59]. The materials used in the manufacture of OA suffer because of the aging process due to the exposure of OA to oral fluids and temperature variations in the oral cavity. OAs physical and chemical properties change, and their efficiency is reduced [82]. Lately, 3D-printed OAs of biocompatible polymers have been preferred because of their proven stability in the oral environment and the possibility of rapidly obtaining a new appliance [82].

In a randomized clinical trial on 26 patients with sleep bruxism comparing the EMG effects of conventional OA with those of 3D printed OA, Bargellini A et al. [56] showed that the 3D printed splints had a more significant impact on electromyographic activity of the masseter muscles related to sleep bruxism, but without influencing the sleep-bruxism index. In the Bargellini et al. study, 3D-printed occlusal appliances controlled better general phasic contractions (grinding) over time but increased tonic contractions (clenching). In the present study, 3D printed occlusal appliances reduced both types of sleep contractions of the masseter muscle, tonic and phasic, in the moderate sleep bruxism group. Regarding the electromyographic results obtained after wearing the splint, they differ depending on many factors, including the design of the splint, the material from which it is made, the manufacturing process (conventional, digitally milled or printed, etc.), the time and frequency of wearing the splint [56]. According to the present study, the effects of 3D printed occlusal appliances also differ depending on the severity of sleep bruxism, since in the low sleep bruxism group, the effects were light, and in the moderate sleep bruxism group, the effects were significant in all sleep and awake-bruxism indices and masseter activity indices. Also, the number of participants with awake bruxism decreased after OA use for 3 months in both groups.

According to the conclusions of Thymi et al. [66], there are necessary studies with portable sEMG devices in the natural ambiance of the person diagnosed with bruxism. There are a few studies with dia-BRUXO devices [67,68], and the cut-off values for sleep-bruxism and awake-bruxism parameters were not established with certainty by the producers. Producers on the device site list some approximative values for sleep-bruxism and awake-bruxism indices but without a scientific certification (unpublished results 75). Even so, the present study’s results show values similar to that of the producer for sleep bruxism and sleep masseter activity, with S-BPI having a mean value for the low sleep-bruxism group of 0.327% compared with 0.343% of the producer. For awake bruxism, the A-BPI cut-off index was 1.205%. In the present study, 20% of participants from the low sleep-bruxism group had awake bruxism; in the moderate sleep-bruxism group, 45.45% had awake bruxism. In the future, studies are necessary to establish cut-off points for sleep-bruxism and awake-bruxism indices values used by dia-BRUXO devices.

Cid-Verdejo et al. [83] assessed the diagnostic validity of portable EMG devices through a systematic review and meta-analysis. The results of the study showed that portable EMG diagnostic devices have a very good diagnostic capacity, with high sensitivity and specificity compared to polysomnography. The evaluation of masseter muscle activity with sEMG for a period of 24 h in subjects with sleep and awake bruxism has many advantages compared with other types of evaluation, like chair-side EMG or polysomnography. First, it should be noted the length of period of evaluation, that achieve 24 h, that gives the possibility to evaluate both bruxism in sleep time and awake time. Using the portable sEMG in the personal environment of the subject offers the advantage to skip the longer period of accommodation that appears with other types of EMG, and using only one channel contributes to this objective. Unlike the situation of polysomnography, when the subject must sleep in a determined position, on the back, with a device with one channel sEMG the subject could maintain the personal position of sleep, and muscle ordinary behavior is recorded. On the other hand, there could be argued that a two-channel portable sEMG could be better, and the lack of simultaneous recording of the two masseter muscles could be a limitation for this device.

The results of the present study showed that maxillary stabilization occlusal appliance 3D printed from hard acrylic resin has a positive effect on sleep-bruxism evolution. After wearing 3 months of an OA, all sleep-bruxism indices for patients from the moderate sleep-bruxism group were lower than the initial values, and the differences were statistically significant. In the low sleep bruxism group, OAs did not significantly influence the EMG activity of the masseter muscle and sleep-bruxism indices, and the number of events per hour of sleep remained constant. As for awake-bruxism indices, they were lowered in most participants compared to the initial values, but the differences were not statistically significant. Overall, according to the results provided by the Mann–Whitney U test and Wilcoxon signed-rank test, there was a statistically significant change in masseter electromyographic activity and bruxism indices after wearing the OA for three months in patients diagnosed with bruxism; thus, the null hypothesis was rejected.

According to the G*Power 3.1.9.7 test, Heinrich Heine University Düsseldorf, Germany, for assessing the minimum number of subjects included in the study for the results to be correct or valid (20 subjects), we estimated that a total of 21 subjects remaining in the study until the end, was relevant for the obtained results.

The limitations of this study are related to the lack of possibility of a standardized approach to appreciating bruxism indices with one-channel sEMG devices. For the dia-Bruxo device, no studies have been published yet to establish the cut-off criteria for bruxism indices. Perhaps a device with channels on both masseters would have given more data related to the activity of the mobilizing muscles of the mandible, especially during grinding. However, it was not a dominant activity.

## 5. Conclusions

sEMG has proven to be an effective diagnostic method for both sleep and awake bruxism, and it has applications in research and current practice. A maxillary occlusal appliance 3D printed in hard ester-based photopolymer resin has significantly lowered sleep-bruxism indices in moderate sleep-bruxism subjects. In low sleep-bruxism subjects, sleep-bruxism indices have decreased but not significantly.

For the moderate sleep-bruxism subjects’ group, the decrease in masseter muscle activity was carried out on behalf of SB-BWI, SB-BPI, which is translated by the decline in the amplitude of muscle contractions. The number of sleep-bruxism events per hour of sleep and the number of clenching episodes per hour of sleep also decreased. Awake bruxism indices were lowered in most subjects who participated in the study. Still, not significantly, mainly by reducing AB-BTI, is the duration of muscle contraction time because of bruxism awareness.

For the low sleep-bruxism group, the indices of sleep and awake bruxism decreased below the reference value given by the manufacturer. Still, they were statistically insignificant compared to the values initially recorded at the beginning of the study. Awake-bruxism indices were lowered not significantly in most of the subjects by decreasing all bruxism indices.

These findings are significant for dental practitioners, especially for patients undergoing extensive oral rehabilitation treatments and having had dental restoration accidents. Recording an increased activity of the masticatory muscles will guide practitioners toward realizing an occlusal splint that will reduce muscle activity and protect the restorations from excessive occlusal forces.

## Figures and Tables

**Table 1:** Study groups with sleep duration, awake duration, detachment duration, and number of sleep-bruxism episodes before and after wearing OA for 3 months.

Parameter	R1 *				R2 *			
	Min	Max	Median	Mean ± SD	Min	Max	Median	Mean ± SD
Low sleep bruxism								
Hours of sleep	3.80	9.40	6.95 (6.25–7.85)	6.930 ± 1.470	5.70	9.50	7.10 (6.92–8.70)	7.510 ± 1.164
Sleep duration	16.70	39.20	29.05 (26.15–32.70)	29.000 ± 5.919	24.00	39.60	29.75 (28.90–36.45)	31.400 ± 4.817
Awake duration	60.80	83.30	70.95 (67.30–73.85)	71.010 ± 5.915	60.40	76.00	70.25 (63.55–71.10)	68.600 ± 4.817
Detachment duration	0.00	7.00	0.000 (0.00–0.00)	0.700 ± 2.214	0.00	16.40	0.00 (0.00–0.00)	2.510 ± 5.152
Number of SB episodes/h	2.00	3.70	2.650 (2.45–3.37)	2.850 ± 0.568	0.00	9.30	2.15 (1.07–4.07)	2.850 ± 2.738
Moderate sleep bruxism								
Hours of sleep	6.30	9.00	7.30 (6.30–8.50)	7.445 ± 1.026	5.00	9.00	7.200 (5.70–8.00)	7.055 ± 1.303
Sleep duration	26.40	37.50	30.50 (26.60–35.30)	31.100 ± 4.216	20.80	37.50	30.200 (25.00–33.30)	29.745 ± 5.160
Awake duration	62.50	73.60	69.50 (64.70–73.40)	68.900 ± 4.216	62.50	79.20	69.800 (66.70–75.00)	70.255 ± 5.160
Detachment duration	0.00	7.50	0.00 (0.00–0.00)	1.264 ± 2.822	0.00	17.40	0.400 (0.00–7.40)	4.018 ± 5.802
Number of SB episodes/h	4.10	11.00	5.50 (4.20–8.20)	6.159 ± 2.271	0.00	9.20	1.900 (1.30–6.75)	3.370 ± 3.011

* R1 = first session of recording sEMG; R2 = second session of recording sEMG.

**Table 2:** dia-BRUXO parameters (sleep and awake indices) for all participants.

Parameter	R1				R2					Diff (R1 − R2)		*p* *
	Min	Max	Median	Mean ± SD	Min	Max	Median	Mean ± SD	#	Decrease %	Increase %	
SB-BWI	0.100	1.249	0.310 (0.223–0.624)	0.439 ± 0.311	0.000	1.212	0.213 (0.116–0.483)	0.333 ± 0.310	↓	16 (76.19%)	5 (23.81%)	0.030 **
SB-BTI	0.185	2.474	0.444 (0.260–0.706)	0.587 ± 0.514	0.000	1.059	0.363 (0.146–0.691)	0.407 ± 0.324	↓	15 (71.43%)	6 (28.57%)	0.065
SB-BPI	0.178	2.066	0.421 (0.255–0.665)	0.534 ± 0.43	0.000	1.110	0.309 (0.136–0.630)	0.38 ± 0.309	↓	15 (71.43%)	6 (28.57%)	0.037 **
No events/h	2.000	11.000	4.100 (2.650–5.675)	4.583 ± 2.365	0.000	9.300	1.900 (1.350–4.500)	3.122 ± 2.824	↓	14 (66.67%)	7 (33.33%)	0.037 **
SB-clenching	8.000	94.000	22 (15.000–37.500)	29.095 ± 19.131	0.000	75.000	11 (7.000–31.500)	20.571 ± 22.198	↓	17 (80.95%)	4 (19.05%)	0.052
SB-grinding	0.000	23.000	3.000 (1.000–6.000)	4.429 ± 5.776	0.000	16.000	1 (0–6)	3.429 ± 4.833	↓	17 (80.95%)	4 (19.05%)	0.412
SB-others	0.000	2.000	0 (0–0)	0.095 ± 0.436	0.000	0.000	0 (0–0)	0 ± 0	↓	21 (100%)	0 (0%)	0.317
S-MWI	0.261	5.703	0.558 (0.419–0.798)	1.019 ± 1.446	0.090	3.104	0.421 (0.284–0.803)	0.641 ± 0.659	↓	13 (61.90%)	8 (38.10%)	0.375
S-MTI	0.999	30.011	2.007 (1.432–3.011)	4.07 ± 6.56	0.428	29.700	1.752 (1.178–3.304)	3.554 ± 6.25	↓	14 (66.67%)	7 (33.33%)	0.357
S-MPI	0.760	21.908	1.538 (1.098–2.245)	3.044 ± 4.833	0.315	20.835	1.123 (0.847–2.503)	2.574 ± 4.38	↓	14 (66.67%)	7 (33.33%)	0.357
AB-BWI	0.059	9.164	0.607 (0.333–1.056)	1.093 ± 1.907	0.109	3.271	0.595 (0.265–1.241)	0.83 ± 0.791	↓	13 (61.90%)	8 (38.10%)	0.639
AB-BTI	0.064	5.449	0.733 (0.373–1.511)	1.172 ± 1.22	0.109	3.948	0.908 (0.313–1.178)	1.093 ± 1.057	↓	13 (61.90%)	8 (38.10%)	0.664
AB-BPI	0.063	6.687	0.71 (0.376–1.325)	1.146 ± 1.418	0.109	3.722	0.79 (0.284–1.198)	0.998 ± 0.961	↓	13 (61.90%)	8 (38.10%)	0.590
AB-clenching	13.000	404.000	106.000 (54.00–157.00)	116.238 ± 89.009	18.000	406.000	68 (31.500–151.000)	108.762 ± 95.584	↓	13 (61.90%)	8 (38.10%)	0.394
AB-grinding	0.000	177.000	7.000 (2.000–30.5000)	29 ± 47.445	0.000	122.000	10 (3.500–24.000)	23.476 ± 34.744	↓	11 (52.38%)	10 (47.62%)	0.931
AB-others	0.000	21.000	0 (0–0)	1.571 ± 5.163	0.000	0.000	0 (0–0)	0 ± 0	↓	21 (100%)	0 (0%)	0.180
A-MWI	1.365	6.731	3.567 (2.328–4.173)	3.571 ± 1.535	1.420	8.829	2.902 (2.310–4.614)	3.63 ± 1.968	↑	12 (57.14%)	9 (42.86%)	0.768
A-MTI	5.410	38.626	15.69 (9.940–20.673)	16.584 ± 8.279	5.948	39.532	13.518 (9.917–21.655)	16.215 ± 8.659	↓	13 (61.90%)	8 (38.10%)	0.741
A-MPI	4.070	27.915	11.711 (7.403–15.172)	12.246 ± 5.985	4.447	28.768	9.882 (7.801–15.896)	12.103 ± 6.326	↓	12 (57.14%)	9 (42.86%)	0.741
MP	132.000	405.000	337 (278–378)	318.524 ± 74.762	207.000	409.000	325 (293–384.5.00)	329.714 ± 53.64	↑	11 (52.38%)	10 (47.62%)	0.958

* Wilcoxon signed-rank test. ** Statistically significant values. # Evolution trends for bruxism-specific activity, measured by numeric indices.

**Table 3:** dia-BRUXO parameters for participants with low sleep bruxism.

Parameter			R1		R2					Diff (R1 − R2)		*p* *
	Min	Max	Median	Mean ± SD	Min	Max	Median	Mean ± SD	#	Decrease %	Increase %	
SB-BWI	0.1	0.638	0.243 (0.166–0.283)	0.256 ± 0.148	0	1.212	0.174 (0.067–0.411)	0.304 ± 0.361	↑	3 (30%)	7 (70%)	0.575
SB-BTI	0.185	1.249	0.260 (0.200–0.410)	0.373 ± 0.321	0	1.059	0.24 (0.101–0.578)	0.371 ± 0.385	↓	7 (70%)	3 (30%)	0.415
SB-BPI	0.178	0.978	0.255 (0.184–0.367)	0.327 ± 0.242	0	1.11	0.226 (0.09–0.517)	0.352 ± 0.367	↑	3 (30%)	7 (70%)	0.575
No events/h	2	3.7	2.650 (2.450–3.375)	2.850 ± 0.568	0	9.3	2.15 (1.075–4.075)	2.850 ± 2.738	=	6 (60%)	4 (40%)	0.540
SB-clenching	8	32	15 (12.500–22.000)	17.000 ± 6.960	0	75	11 (7.00–31.75)	20.100 ± 22.398	↑	7 (70%)	3 (30%)	0.859
SB-grinding	0	16	1.5 (1–3)	3.000 ± 4.667	0	16	0.5 (0–5)	3.400 ± 6.168	↑	9 (90%)	1 (10%)	0.228
SB-others	0	0	0 (0–0)	0 ± 0	0	0	0 (0–0)	0 ± 0	=	10 (100%)	0 (0%)	-
S-MWI	0.261	4.807	0.424 (0.286–0.589)	0.842 ± 1.399	0.09	1.487	0.377 (0.197–0.850)	0.533 ± 0.445	↓	6 (60%)	4 (40%)	0.878
S-MTI	0.999	13.911	1.495 (1.081–2.302)	2.760 ± 3.951	0.428	7.726	1.319 (0.856–3.951)	2.496 ± 2.445	↓	6 (60%)	4 (40%)	0.959
S-MPI	0.76	10.876	1.14 (0.815–1.731)	2.120 ± 3.100	0.315	5.646	1.01 (0.637–2.950)	1.832 ± 1.776	↓	6 (60%)	4 (40%)	0.959
AB-BWI	0.059	1.682	0.41 (0.284–0.816)	0.578 ± 0.470	0.109	1.212	0.277 (0.191–0.863)	0.501 ± 0.392	↓	7 (70%)	3 (30%)	0.241
AB-BTI	0.064	2.082	0.602 (0.313–1.094)	0.756 ± 0.618	0.109	1.228	0.478 (0.237–1.010)	0.607 ± 0.398	↓	7 (70%)	3 (30%)	0.386
AB-BPI	0.063	1.948	0.591 (0.304–0.944)	0.697 ± 0.558	0.109	1.222	0.411 (0.220–0.938)	0.573 ± 0.392	↓	7 (70%)	3 (30%)	0.333
AB-clenching	13	243	73.500 (56.250–152)	100.300 ± 68.769	24	173	51 (29.25–143.00)	81.700 ± 60.312	↓	7 (70%)	3 (30%)	0.139
AB-grinding	0	41	4.5 (1.000–16.750)	10.600 ± 14.120	0	16	4.5 (3.00–12.25)	6.500 ± 5.233	↓	7 (70%)	3 (30%)	0.441
AB-others	0	21	0 (0–0)	2.100 ± 6.641	0	0	0 (0–0)	0 ± 0	↓	10 (100%)	0 (0%)	0.317
A-MWI	1.365	4.607	3.141 (1.707–4.155)	3.023 ± 1.171	1.446	8.829	3.049 (2.322–4.291)	3.605 ± 2.114	↑	5 (50%)	5 (50%)	0.508
A-MTI	5.41	21.336	15.224 (6.732–19.269)	13.852 ± 5.854	5.948	26.74	13.902 (10.037–20.557)	15.389 ± 6.887	↑	6 (60%)	4 (40%)	0.721
A-MPI	4.07	15.604	11.309 (5.054–13.977)	10.243 ± 4.253	4.447	20.77	10.22 (8.130–15.088)	11.566 ± 5.151	↑	5 (50%)	5 (50%)	0.646
MP	271	405	336 (293.50–371.25)	334.300 ± 46.390	256	399	332.5 (312.750–384.250)	337.900 ± 42.969	↑	5 (50%)	5 (50%)	0.575

* Wilcoxon signed-rank test. # Evolution trends for bruxism-specific activity, measured by numeric indices.

**Table 4:** dia-BRUXO parameters for participants with moderate sleep bruxism.

Parameter	R1				R2					Diff (R1 − R2)		*p* *
	Min	Max	Median	Mean ± SD	Min	Max	Median	Mean ± SD	#	Decrease %	Increase %	
SB-BWI	0.212	1.249	0.553 (0.356–0.761)	0.606 ± 0.331	0	0.832	0.336 (0.116–0.677)	0.359 ± 0.271	↓	9 (81.82%)	2 (18.18%)	0.016 **
SB-BTI	0.384	2.474	0.593 (0.453–0.900)	0.781 ± 0.591	0	0.81	0.466 (0.149–0.714)	0.439 ± 0.273	↓	8 (72.73%)	3 (27.27%)	0.062
SB-BPI	0.359	2.066	0.534 (0.429–0.869)	0.722 ± 0.485	0	0.77	0.423 (0.138–0.702)	0.405 ± 0.261	↓	8 (72.73%)	3 (27.27%)	0.029 **
No events/h	4.1	11	5.5 (4.200–8.200)	6.159 ± 2.271	0	9.2	1.9 (1.300–6.750)	3.37 ± 3.011	↓	8 (72.73%)	3 (27.27%)	0.026 **
SB-clenching	20	94	37 (28–44)	40.091 ± 20.226	0	73	11 (7–32)	21 ± 23.1	↓	10 (90.91%)	1 (9.09%)	0.010 **
SB-grinding	0	23	4 (1–6)	5.727 ± 6.574	0	10	3 (0–6)	3.455 ± 3.532	↓	8 (72.73%)	3 (27.27%)	0.499
SB-others	0	2	0 (0–0)	0.182 ± 0.603	0	0	0 (0–0)	0 ± 0	↓	11 (100%)	0 (0%)	0.317
S-MWI	0.418	5.703	0.637 (0.502–0.888)	1.179 ± 1.536	0.118	3.104	0.433 (0.329–0.804)	0.74 ± 0.817	↓	7 (63.64%)	4 (36.36%)	0.374
S-MTI	1.41	30.011	2.709 (1.992–3.800)	5.262 ± 8.291	0.634	29.7	1.752 (1.344–3.265)	4.516 ± 8.402	↓	8 (72.73%)	3 (27.27%)	0.286
S-MPI	1.079	21.908	2.019 (1.495–2.829)	3.884 ± 6.036	0.462	20.835	1.357 (1.046–2.445)	3.248 ± 5.872	↓	8 (72.73%)	3 (27.27%)	0.286
AB-BWI	0.094	9.164	0.937 (0.411–1.462)	1.562 ± 2.562	0.146	3.271	0.827 (0.323–1.438)	1.128 ± 0.952	↓	6 (54.55%)	5 (45.45%)	0.929
AB-BTI	0.242	5.449	1.115 (0.552–2.409)	1.549 ± 1.517	0.191	3.948	1.020 (0.449–2.685)	1.535 ± 1.281	↓	6 (54.55%)	5 (45.45%)	1
AB-BPI	0.217	6.687	1.056 (0.505–2.108)	1.554 ± 1.832	0.176	3.722	0.836 (0.407–2.254)	1.383 ± 1.168	↑	6 (54.55%)	5 (45.45%)	0.929
AB-clenching	17	404	121 (47.00–172.00)	130.727 ± 105.377	18	406	97 (52–221)	133.364 ± 116.623	↑	6 (54.55%)	5 (45.45%)	1
AB-grinding	1	177	23 (3–109)	45.727 ± 60.632	2	122	15 (9–86)	38.909 ± 42.889	↓	4 (36.36%)	7 (63.64%)	0.721
AB-others	0	12	0 (0–0)	1.091 ± 3.618	0	0	0 (0–0)	0 ± 0	↓	11 (100%)	0 (0%)	0.317
A-MWI	1.571	6.731	3.96 (2.817–5.784)	4.069 ± 1.704	1.42	7.241	2.830 (2.244–5.867)	3.653 ± 1.93	↓	7 (63.64%)	4 (36.36%)	0.286
A-MTI	6.747	38.626	17.475 (12.024–27.371)	19.067 ± 9.592	6.341	39.532	13.216 (8.591–27.232)	16.966 ± 10.294	↓	7 (63.64%)	4 (36.36%)	0.477
A-MPI	5.021	27.915	13.009 (8.955–19.957)	14.068 ± 6.907	4.78	28.768	9.684 (6.475–20.153)	12.59 ± 7.456	↓	7 (63.64%)	4 (36.36%)	0.424
MP	132	398	341 (205–381)	304.182 ± 93.631	207	409	307 (287–400)	322.273 ± 62.967	↑	6 (54.55%)	5 (45.45%)	0.722

* Wilcoxon signed-rank test. ** Statistically significant values. # Evolution trends for bruxism-specific activity, measured by numeric indices.

**Table 5:** dia-BRUXO Recording 1 parameters for participants with low and moderate sleep bruxism.

dia-BRUXO Parameter	Median		Mean ± SD		*p* *
	Low	Moderate	Low	Moderate	
R1_SB-BWI	0.243 (0.166–0.283)	0.553 (0.356–0.761)	0.256 ± 0.148	0.606 ± 0.331	0.002 **
R1_SB-BTI	0.260 (0.200–0.410)	0.593 (0.453–0.900)	0.373 ± 0.321	0.781 ± 0.591	0.003 **
R1_SB-BPI	0.255 (0.184–0.367)	0.534 (0.429–0.869)	0.327 ± 0.242	0.722 ± 0.485	0.001 **
R1_no events/h	2.650 (2.450–3.375)	5.5 (4.200–8.200)	2.85 ± 0.568	6.159 ± 2.271	<0.0001 **
R1_SB-clenching	15 (12.500–22.000)	37 (28–44)	17 ± 6.96	40.091 ± 20.226	<0.0001 **
R1_SB-grinding	1.5 (1.0–3.0)	4 (1–6)	3 ± 4.667	5.727 ± 6.574	0.173
R1_SB-others	0 (0–0)	0 (0–0)	0 ± 0	0.182 ± 0.603	0.756
R1_S-MWI	0.424 (0.286–0.589)	0.637 (0.502–0.888)	0.842 ± 1.399	1.179 ± 1.536	0.02 **
R1_S-MTI	1.495 (1.081–2.302)	2.709 (1.992–3.800)	2.76 ± 3.951	5.262 ± 8.291	0.02 **
R1_S-MPI	1.140 (0.815–1.731)	2.019 (1.495–2.829)	2.12 ± 3.1	3.884 ± 6.036	0.016 **
R1_AB-BWI	0.410 (0.284–0.816)	0.937 (0.411–1.462)	0.578 ± 0.47	1.562 ± 2.562	0.173
R1_AB-BTI	0.602 (0.313–1.094)	1.115 (0.552–2.409)	0.756 ± 0.618	1.549 ± 1.517	0.152
R1_AB-BPI	0.591 (0.304–0.944)	1.056 (0.505–2.108)	0.697 ± 0.558	1.554 ± 1.832	0.173
R1_AB-clenching	73.500 (56.250–152)	121 (47–172)	100.3 ± 68.769	130.727 ± 105.377	0.512
R1_AB-grinding	4.500 (1.000–16.750)	23 (3–109)	10.6 ± 14.12	45.727 ± 60.632	0.152
R1_AB-others	0 (0–0)	0 (0–0)	2.1 ± 6.641	1.091 ± 3.618	0.973
R1_A-MWI	3.141 (1.707–4.155)	3.960 (2.817–5.784)	3.023 ± 1.171	4.069 ± 1.704	0.197
R1_A-MTI	15.224 (6.732–19.269)	17.475 (12.024–27.371)	13.852 ± 5.854	19.067 ± 9.592	0.223
R1_A-MPI	11.309 (5.054–13.977)	13.009 (8.955–19.957)	10.243 ± 4.253	14.068 ± 6.907	0.251
R1_MP	336 (293.50–371.25)	341 (205–381)	334.3 ± 46.39	304.182 ± 93.631	0.809

* Mann–Whitney U test. ** Statistically significant values.

**Table 6:** dia-BRUXO Recording 2 parameters for participants with low and moderate sleep bruxism.

dia-BRUXO Parameter	Median		Mean ± SD		*p* *
	Low	Moderate	Low	Moderate	
R2_SB-BWI	0.174 (0.067–0.411)	0.336 (0.116–0.677)	0.304 ± 0.361	0.359 ± 0.271	0.468
R2_SB-BTI	0.240 (0.101–0.578)	0.466 (0.149–0.714)	0.371 ± 0.385	0.439 ± 0.273	0.426
R2_SB-BPI	0.226 (0.09–0.517)	0.423 (0.138–0.702)	0.352 ± 0.367	0.405 ± 0.261	0.426
R2_no events/h	2.150 (1.075–4.075)	1.9 (1.30–6.75)	2.85 ± 2.738	3.37 ± 3.011	0.863
R2_SB-clenching	11 (7.00–31.75)	11 (7–32)	20.1 ± 22.398	21 ± 23.1	0.973
R2_SB-grinding	0.5 (0–5)	3 (0–6)	3.4 ± 6.168	3.455 ± 3.532	0.557
R2_SB-others	0 (0–0)	0 (0–0)	0 ± 0	0 ± 0	1
R2_S-MWI	0.377 (0.197–0.850)	0.433 (0.329–0.804)	0.533 ± 0.445	0.74 ± 0.817	0.426
R2_S-MTI	1.319 (0.856–3.951)	1.752 (1.344–3.265)	2.496 ± 2.445	4.516 ± 8.402	0.387
R2_S-MPI	1.010 (0.637–2.950)	1.357 (1.046–2.445)	1.832 ± 1.776	3.248 ± 5.872	0.349
R2_AB-BWI	0.277 (0.191–0.863)	0.827 (0.323–1.438)	0.501 ± 0.392	1.128 ± 0.952	0.061
R2_AB-BTI	0.478 (0.237–1.010)	1.020 (0.449–2.685)	0.607 ± 0.398	1.535 ± 1.281	0.152
R2_AB-BPI	0.411 (0.22–0.938)	0.836 (0.407–2.254)	0.573 ± 0.392	1.383 ± 1.168	0.173
R2_AB-clenching	51 (29.250–143.000)	97 (52–221)	81.7 ± 60.312	133.364 ± 116.623	0.349
R2_AB-grinding	4.5 (3.00–12.25)	15 (9–86)	6.5 ± 5.233	38.909 ± 42.889	0.051
R2_AB-others	0 (0–0)	0 (0–0)	0 ± 0	0 ± 0	1
R2_A-MWI	3.049 (2.322–4.291)	2.830 (2.244–5.867)	3.605 ± 2.114	3.653 ± 1.93	0.973
R2_A-MTI	13.902 (10.037–20.557)	13.216 (8.591–27.232)	15.389 ± 6.887	16.966 ± 10.294	0.973
R2_A-MPI	10.22 (8.130–15.088)	9.684 (6.475–20.153)	11.566 ± 5.151	12.59 ± 7.456	0.863
R2_MP	332.5 (312.75–384.25)	307 (287–400)	337.9 ± 42.969	322.273 ± 62.967	0.605

* Mann–Whitney U test.

**Table 7:** dia-BRUXO differences (Diff) between values corresponding to R1 and R2 parameters for participants with low and moderate sleep bruxism.

dia-BRUXO Parameter	Median		Mean ± SD		*p* *
	Low	Moderate	Low	Moderate	
Diff_SB-BWI	0.060 (−0.113–0.141)	0.212 (0.023–0.413)	−0.048 ± 0.342	0.247 ± 0.343	0.072
Diff_SB-BTI	0.119 (−0.142–0.203)	0.144 (−0.009–0.558)	0.002 ± 0.297	0.342 ± 0.705	0.426
Diff_SB-BPI	0.081 (−0.133–0.174)	0.164 (−0.029–0.429)	−0.025 ± 0.305	0.317 ± 0.57	0.173
Diff_no events/h	0.950 (−1.300–1.900)	3.480 (−0.500–5.300)	0 ± 2.519	2.789 ± 3.244	0.072
Diff_SB-clenching	6 (−16.250–12.000)	21 (7–32)	−3.1 ± 20.899	19.091 ± 16.932	0.013 **
Diff_SB-grinding	1 (0–1.25)	0 (−2.00–6.00)	−0.4 ± 4.526	2.273 ± 8.014	0.705
Diff_SB-others	0 (0–0)	0 (0–0)	0 ± 0	0.182 ± 0.603	0.756
Diff_S-MWI	0.039 (−0.168–0.194)	0.081 (−0.130–0.449)	0.309 ± 1.078	0.439 ± 1.888	0.557
Diff_S-MTI	0.081 (−0.763–0.583)	0.535 (−0.818–1.358)	0.264 ± 2.395	0.746 ± 12.441	0.314
Diff_S-MPI	0.120 (−0.583–0.453)	0.384 (−0.600–1.215)	0.288 ± 1.932	0.636 ± 8.896	0.387
Diff_AB-BWI	0.086 (−0.059–0.174)	0.012 (−0.765–0.264)	0.076 ± 0.193	0.434 ± 2.562	0.387
Diff_AB-BTI	0.073 (−0.177–0.508)	0.041 (−0.651–0.284)	0.149 ± 0.413	0.014 ± 1.374	0.557
Diff_AB-BPI	0.077 (−0.138–0.365)	0.041 (−0.689–0.278)	0.124 ± 0.332	0.17 ± 1.705	0.426
Diff_AB-clenching	16.500 (−8.000–41.250)	12 (−112.000–75.000)	18.6 ± 44.217	−2.636 ± 130.671	0.756
Diff_AB-grinding	1 (−4.25–9.00)	−3 (−13.000–12.000)	4.1 ± 13.788	6.818 ± 45.609	0.314
Diff_AB-others	0 (0–0)	0 (0–0)	2.1 ± 6.641	1.091 ± 3.618	0.973
Diff_A-MWI	−0.002 (−1.605–0.688)	0.668 (−0.748–1.384)	−0.582 ± 1.717	0.416 ± 1.351	0.132
Diff_A-MTI	0.299 (−9.517–5.772)	0.807 (−1.087–6.161)	−1.537 ± 7.618	2.101 ± 8.139	0.512
Diff_A-MPI	0.063 (−7.020–4.107)	0.761 (−0.853–4.256)	−1.324 ± 5.561	1.477 ± 5.8	0.387
Diff_MP	−1 (−30.00–23.00)	8 (−19.000–26.000)	−3.6 ± 35.88	−18.091 ± 99.724	0.654

* Mann–Whitney U test. ** Statistically significant values.

## Data Availability

The authors declare that the data of this research are available from the corresponding authors upon reasonable request.

## Abbreviations

EMG	electromyographic activity
MMA	masticatory muscles activity
SB	sleep bruxism
AB	awake bruxism
BWI	bruxism effort index
BTI	bruxism time index
BPI	bruxism personal index
MWI	muscle effort index
MTI	muscle time index
MPI	muscle activity personal index
OA	occlusal appliance
